# The World Network of Psychiatric Trainees’ Human Rights Curriculum Initiative

**DOI:** 10.1192/j.eurpsy.2023.159

**Published:** 2023-07-19

**Authors:** V. Pereira-Sanchez, NYU

**Affiliations:** Child and Adolescent Psychiatry, New York University Grossman School of Medicine, New York, United States

## Abstract

**Abstract:**

Dr. Pereira-Sanchez, Founder and Executive Director of the World Network of Psychiatric Trainees (WNPT) and responsible for the WNPT Human Rights Curriculum Initiative, will present an overview of the same, which aims at understanding the current state of human rights education for psychiatric trainees across the world and at partnering with relevant organizations to develop international standards on the topic. The talk will highlight pilot results and achievments of the initiative and invite feedback, discussion, and further collaboration.

**Disclosure of Interest:**

None Declared

